# Working Memory as a Candidate Mechanism Linking Aging and the Sense of Agency: A Narrative Review

**DOI:** 10.3390/brainsci16050510

**Published:** 2026-05-10

**Authors:** Toshiya Nezu, Yuji Fujino, Hinayo Kaneko, Tadamitsu Matsuda, Tsubasa Kawasaki, Toshiyuki Fujiwara

**Affiliations:** 1 Department of Physical Therapy, Graduate School of Health Science, Juntendo University, Tokyo 113-8421, Japan; nezu.toshiyapt@gmail.com (T.N.); t.matsuda.ye@juntendo.ac.jp (T.M.); t-fujiwara@juntendo.ac.jp (T.F.); 2 Department of Rehabilitation, Shuuwa General Hospital, Saitama 344-0035, Japan; 3 Department of Rehabilitation, Saitama Citizens Medical Center, Saitama 331-0054, Japan; 4 Department of Physical Therapy, School of Health Sciences, Tokyo International University, Saitama 350-1197, Japan; kawasaki.283@gmail.com; 5 Department of Rehabilitation Medicine, Graduate School of Medicine, Juntendo University, Tokyo 113-8421, Japan

**Keywords:** sense of agency, working memory, cue integration, intentional binding, cognitive load, aging

## Abstract

**Highlights:**

**What are the main findings?**

- The sense of agency is conceptualized as a multi-component process involving the integration of predictive, sensory, and contextual cues.
- Working-memory-related processes may support prediction maintenance, action–outcome comparison, and cue weighting, helping to explain non-uniform age-related changes in agency.

**What are the implications of the main findings?**

- Evidence linking working memory to the sense of agency remains indirect and is often confounded by attention and cognitive-control demands.
- Future research should integrate implicit and explicit agency measures with longitudinal, ecological, and interventional designs to clarify mechanisms linking aging, working memory, and agency.

**Abstract:**

Sense of agency (SoA) refers to the experience or belief that one’s own actions directly lead to a particular event in the body or external world. SoA does not arise from a single mechanism; rather, evidence suggests the integration of predictive, sensory, and contextual cues whose relative weighting may vary across tasks and individuals. Since this process requires the maintenance, comparison, and updating of action-related information, working memory-related processes have been proposed as a candidate contributor to selected components of agency. At the same time, aging is associated with alterations in temporal processing, sensory precision, executive control, and other functions that may change the use of agency-related cues. However, the extent to which age-related differences in SoA can be explained by working memory-related processes remains undefined. This narrative review examines the theoretical and empirical basis for considering working memory as a candidate mechanism linking aging and SoA. Specifically, it discusses major theoretical frameworks of agency, measurement approaches, evidence on aging-related differences in SoA, and studies suggesting that cognitive load- and working memory-related factors may modulate agency-related processing. Current evidence is suggestive, although it remains indirect, highlighting key methodological limitations and outlining priorities for future research to clarify how aging- and working-memory-related processes may shape different components of agency.

## 1. Introduction

The feeling of acting according to our own will and producing outcomes through our actions in daily life may appear self-evident; however, it is, in fact, the product of highly complex information processing. This sense of “causing one’s own actions” is known as the Sense of Agency (SoA) and constitutes an important area of research in cognitive neuroscience [1]. SoA refers to the subjective feeling that one is the agent who has caused physical changes in one’s body or the environment [2]. A typical example is the realization that “my action is the cause” when a light turns on after flipping a switch or when a cursor moves on a screen in response to operating a computer mouse.

The comparator model, proposed by Frith et al., is widely considered a representative framework for explaining SoA [3,4]. According to this model, before initiating a movement, the brain generates an “efference copy,” a duplicate of the motor command (effector command), from which it derives a predicted sensation (internal cue). After the actual movement occurs, sensory feedback from external sources—including visual, auditory, and somatosensory information—is received. These two are compared, and it is believed that the higher the degree of match, the stronger the SoA, or the feeling that one is “acting as intended” [5,6]. Furthermore, recently, the perspective of “cue integration,” which involves the Bayesian integration of information from multiple modalities, including vision, hearing, and somatosensation, has been gaining attention. This indicates that SoA may be adjusted by comprehensively evaluating the reliability of diverse information sources rather than relying on a single sensation [6,7,8].

Furthermore, SoA measurement is broadly categorized into explicit SoA, which involves directly reporting one’s subjective Feeling of Agency (FoA), and implicit SoA, which is indirectly assessed through factors such as temporal or spatial discrepancies [1,9]. Using these methods, numerous studies have been conducted, including those with [10] and without [11] physical activity, as well as those investigating its neural mechanisms [12,13]. In addition to research on healthy adults, clinical studies on patients with schizophrenia [14,15] and post-stroke patients [16,17] have suggested that a breakdown of SoA is deeply involved in their symptoms and the success or failure of rehabilitation. Moreover, in the context of recent virtual reality (VR) and teleoperation technologies, how stably a user can feel SoA affects the sense of control and performance, prompting active research in engineering, medicine, and psychology [18,19].

SoA is susceptible to individual differences and age-related changes, and a growing number of studies have specifically examined age-related differences [20,21]. Developmental studies have shown that the temporal window of SoA differs between school-age children and young adults [20], while lifespan studies suggest that agency-related temporal processing may also differ in older adults [21]. The temporal window of SoA refers to the range of temporal discrepancy between a self-generated movement and external feedback, such as visual or auditory feedback, within which SoA is maintained before it begins to diminish [20]. In older adults, SoA changes due to age-related declines in frontal lobe function, attentional, and action-selection abilities [21]. Indeed, cases have been reported in which SoA is less likely to decline despite delays in visual feedback, and others in which it is more likely to diminish [21,22,23]. According to research by Metcalfe et al. [24] and Cavazzana et al. [21], older adults rely more strongly on internal cues. Consequently, their SoA is less likely to be disturbed by slight delays in external feedback. However, in cases of significant frontal lobe decline, SoA may be less likely to emerge.

Recently, working memory (WM) has gained attention as a cognitive mechanism underlying these age-related and individual differences [25]. WM refers to the function of temporarily holding and manipulating information necessary for complex tasks, including learning, reasoning, and language comprehension. It is considered a higher-order cognitive function composed of multiple subsystems, as described in Baddeley’s multi-component model [26]. The process of holding a prediction and appropriately comparing it with actual sensory feedback is essential for establishing SoA, and WM resources are likely involved in this process. For example, when WM resources are limited by physical exertion or acute psychosocial stress, the temporal precision of multisensory integration deteriorates, and implicit and explicit measures of agency are weakened [27,28,29,30,31], consistent with predictive-coding accounts in which attention optimizes the precision of sensory prediction errors [32].

The decline in WM function with aging is widely known [33,34,35,36,37]; however, it remains unclear to what extent this decline in WM capacity affects the process of SoA, particularly regarding comparing internal predictions with external feedback. This review re-examines the role of WM in the emergence of SoA and organizes previous findings around the central question of how age-related changes in SoA relate to the decline in WM function. Accordingly, the aim of this narrative literature review is to clarify the theoretical and empirical basis for a possible link between age-related changes in SoA and declines in WM function. First, it provides an overview of the theoretical background and measurement methods of SoA; second, it discusses factors contributing to age-related changes in SoA; and finally, it considers the role of WM and future research challenges.

## 2. Methods

Using online databases, including PubMed and ScienceDirect, this narrative review identified relevant literature. Searches were conducted iteratively during this review, and the final search update was performed on 9 April 2026. Representative search terms included sense of agency, agency judgment, intentional binding, working memory, cognitive load, aging, older adults, and cue integration. Combinations of these terms were also used, including “sense of agency and aging,” “sense of agency and working memory,” “intentional binding and older adults,” and “agency judgment and cognitive load.” The review focused primarily on peer-reviewed articles published in English. Additional relevant studies were identified via manual screening of the reference lists of key articles.

This review was narrative rather than systematic; therefore, studies were selected based on conceptual relevance and methodological informativeness to the central aim of the review, namely, to evaluate how working memory-related processes contribute to age-related variation in SoA. Conceptual studies were included when they were necessary to frame major theoretical accounts of agency, whereas empirical studies were prioritized when they directly informed at least one of the following domains: measurement of the sense of agency, aging-related differences in agency, working-memory- or cognitive-load-related modulation of agency, and neural mechanisms relevant to prediction, comparison, and cue integration. When multiple studies addressed closely overlapping paradigms or research questions, priority was given to those that were methodologically representative, conceptually influential, or particularly informative regarding interpretive limitations. Table 1 presents a structured summary of key empirical studies considered central to the present review.

Participant descriptors were standardized across studies for readability and consistency. Age information is presented as reported in the original articles. Full age ranges are shown; when age ranges were not reported, mean age and standard deviation are provided. “Healthy adults” refers to adult participants described in the original studies as neurologically and psychiatrically healthy and does not imply that identical screening procedures were used across all studies.

## 3. Theoretical Foundations of SoA

SoA, the subjective experience that one’s own actions produce outcomes, is widely assumed to be supported by both internal cues, including predictive signals derived from motor control, and external cues, such as visual/auditory feedback, as well as contextual and belief-related information. These multiple cues are believed to be weighted according to their relative reliability in a given situation and integrated into a final agency judgment [8]. Two representative frameworks for explaining SoA are as follows: (i) the comparator model, grounded in mechanisms of motor control, and (ii) apparent mental causation, which explains agency as an inference based on the correspondence between thoughts and actions. However, these accounts are better understood not as mutually exclusive theories but as frameworks whose relative contributions vary depending on the processing level they emphasize (a sensorimotor level based on prediction error vs. an inferential level guided by context and beliefs) and the type of judgment required by the task (an immediate experience vs. a retrospective evaluation). This perspective aligns with integrative accounts, including the two-step theory and explanations that incorporate interactions between prediction and postdiction [9,42].

In this section, we outline this theoretical trajectory in the following order: the motor-control-based comparator model, the belief-based apparent mental causation account, and then integrative approaches that bridge the two, namely the two-step theory and cue integration.

### 3.1. Comparator Model

One of the most influential theories of SoA is the comparator model, which originated from computational models of motor control [4,43]. In this framework, predictive signals accompanying motor commands (a forward model based on an efference copy) are compared with actual sensory consequences (visual, auditory, and somatosensory feedback), and the resulting mismatch (prediction error) is assumed to adjust self-attribution [4,5,43].

A key strength of this model is that it offers testable predictions: behavioral studies consistently show that experimentally manipulable errors, including distortions or delays in external feedback, systematically reduce SoA (e.g., by manipulating visual feedback or inducing mismatches between predicted and actual outcomes) [5,44,45,46]. Accordingly, the accuracy of motor prediction is critical in this account, and larger prediction errors are suggested to lead to reduced SoA [46].

One prominent phenomenon supporting this model is sensory attenuation [47]. Sensory attenuation refers to the observation that self-generated sensory inputs (e.g., trying to tickle oneself) are perceived as weaker than externally generated sensory inputs of the same physical intensity. This effect is explained by motor predictions that “cancel” or downregulate the sensory consequences of one’s own actions [47,48,49]. However, recent research has re-examined whether sensory attenuation can be explained solely by such a simple cancellation mechanism. Although many findings support a contribution of predictive processes, additional factors (e.g., postdictive interpretation or expectation) may also play a role depending on tasks and contexts [9,50,51,52]. Therefore, sensory attenuation remains a canonical example showing that prediction can modulate perception; conversely, it is important not to overgeneralize it as a single process or fully specific marker [47].

Importantly, the comparator model primarily accounts for processing at a relatively low sensorimotor level and has been criticized for its limited ability to capture higher-level cognitive influences on SoA (e.g., prior knowledge, context, and beliefs). For instance, reports that SoA can vary when action selection is manipulated by subliminal primes [53], among other findings, suggest that the comparator model alone is insufficient [51,54,55]. Another crucial issue is that implicit measures (e.g., sensory attenuation and intentional binding [IB]) and explicit measures (e.g., self-attribution ratings) do not necessarily converge. Specifically, the comparator model may more strongly capture implicit, sensorimotor-leaning components, and it may not map one-to-one onto explicit agency judgment [39]. Furthermore, noninvasive brain stimulation targeting the pre-supplementary motor area (SMA) and the inferior parietal cortex has been reported to modulate SoA-related measures (e.g., IB and prospective SoA), suggesting a causal contribution of frontoparietal networks to SoA [56,57,58,59] (see Section 7.1 for methodological limitations and interpretive issues in stimulation studies). Overall, this multi-layered view motivates frameworks that treat SoA not as determined solely by prediction error but as arising from the integration of multiple cues—prediction, sensory inputs, and contextual beliefs—weighted by their reliability (i.e., cue integration) [8]. This consideration partly motivated the development of subsequent theoretical models.

### 3.2. Theory of Apparent Mental Causation

A prominent account that emphasizes higher-order cognitive aspects that are difficult to explain within the comparator model is the theory of apparent mental causation proposed by Wegner et al. [60]. This theory argues that SoA does not arise directly from sensorimotor matching; rather, it is formed through an inferential process that interprets the causal relation between a preceding intention/thought and an observed outcome.

Specifically, SoA is more likely to occur when the following three conditions are met: the thought precedes the action (priority), the thought is consistent with the action/outcome (consistency), and no other plausible cause is available (exclusivity) [60,61]. Simultaneously, this inferential framework alone has difficulty explaining fluctuations in SoA under conditions where prediction error and sensorimotor constraints play major roles, implying the need for integrative models that connect low-level error processing to high-level interpretive processes. In particular, prediction-based sensorimotor signatures (e.g., sensory attenuation) are difficult to fully replace with post hoc inference alone [47].

In Wegner’s experiments supporting this theory, participants reported an illusory SoA over another person’s arm movements when they received primes that were consistent with the observed movements [62]. This vicarious agency over others’ actions is a representative example showing that context and inference can substantially reshape SoA [63]. Similarly, Aarts et al. demonstrated that merely priming information about action outcomes can enhance SoA for those outcomes regardless of whether the participant actually executed the action [64]. Moreover, manipulating causal beliefs alone changes IB, an implicit measure of SoA, suggesting that belief-level information can influence processes as low-level as time perception [65].

Therefore, this theory can explain the flexibility of SoA as a function of beliefs and social context. However, this framework alone cannot account for embodied, sensorimotor-level phenomena, including prediction error and sensory attenuation. Clinical research has also indicated that impairments in predicting action outcomes can coexist with context-dependent abnormalities of agency, implying potential dissociations between predictive and postdictive/inferential components; thus, specifying their interaction and weighting is more informative than reducing SoA to either prediction or inference alone [55]. Overall, while apparent mental causation explains context- and belief-driven changes in SoA, it remains unresolved to what extent it can unify prediction-dependent measures (e.g., sensory attenuation) and the dissociation between implicit and explicit measures.

Accordingly, apparent mental causation is useful for explaining aspects of SoA that are closer to Judgment of Agency (JoA); however, it is insufficient as a standalone theory for FoA. These considerations highlight the need for a framework that quantifies when external cues act, at which stage (FoA vs. JoA), and with what weight, based on cue reliability.

### 3.3. Integrative Approaches: Two-Step Theory and Cue Integration

The two theories reviewed above—the comparator model and apparent mental causation—capture important aspects of SoA but also have complementary limitations. Integrative approaches that aim to bridge low-level sensorimotor information and high-level conceptual judgment within a single framework include the two-step account and cue integration.

The two-step account assumes that SoA is generated through two qualitatively different processing stages: a prereflective, non-conceptual FoA, and a reflective, belief-based JoA [42]. In the first stage, FoA is formed from multiple sources of information, including motor predictions and prediction errors; proprioceptive, visual, and auditory feedback; action-selection fluency; and broader environmental statistics and temporal delays [9]. These cues are dynamically integrated and weighted according to their reliability [8,9]. In the second stage, the FoA formed in the first stage is re-evaluated through higher-order conceptual modules involving beliefs, context, and knowledge, yielding a consciously reportable JoA [42]. This hierarchical framework has a major theoretical advantage because it can account for contextual/belief influences and clinically observed alterations of SoA as biases in the weighting assigned to particular cues.

This organization also corresponds to the later distinction between prospective and retrospective cues and implies that FoA and JoA are not strictly independent layers but can influence each other in context and with learning.

For a review, it is also important to clarify which measures tend to reflect which stage. Generally, sensory attenuation and IB are frequently considered relatively FoA-leaning measures because they are closely linked to sensorimotor prediction and error processing, whereas self-attribution or agency ratings are more strongly influenced by task understanding, beliefs, and contextual interpretation and consequently tend to be JoA-leaning [39]. Therefore, dissociations between implicit and explicit measures need not indicate a “contradiction” but may be treated coherently as reflecting differences in stage (FoA vs. JoA) and/or cue weighting [8,42].

From a temporal perspective, SoA formation depends on both prospective cues available before the outcome and retrospective cues available after the outcome. Particularly, prospective cues, including action-selection fluency, strongly influence FoA formation [66,67]. In this organization, prospective cues primarily contribute to FoA estimation near action selection and prediction, whereas retrospective cues primarily contribute to JoA formation and revision through outcome interpretation [7]. Experimentally, manipulating action-selection fluency via subliminal priming or task conflict causes systematic changes in subjective SoA even when outcome predictability is held constant [53,67]. Moreover, the relative weights of prospective and retrospective cues can shift dynamically as learning proceeds [68,69], supporting the view that SoA is not fixed but is adaptively estimated depending on context. The finding that high-level contextual cues, including causal beliefs, can influence low-level processing such as time perception [65] further suggests a complex interaction rather than strict independence between FoA and JoA.

The cue integration approach computationally formalizes—particularly for the first stage of the two-step account—the information integration process using Bayesian inference [8]. In this framework, SoA is estimated by integrating internal cues such as motor prediction with external cues, including outcomes and context [8]. Cue weights are optimized based on their reliability, and SoA emerges as the final estimate [7,8,41,63,70]. Consistent with this view, when internal cues are unreliable (e.g., sensory information is ambiguous), the weight of external cues increases, and SoA can be complemented or reconstructed via retrospective inference [40].

External cues may also include motivational information such as outcome value (reward/punishment) and action–outcome contingency (control/contingency), which can modulate SoA-related measures, including IB and self-attribution judgments [71,72,73,74]. However, general theories of motivational optimization and motor learning should be treated separately, as they suggest relevance but do not constitute direct tests using SoA measures [75,76]. Moreover, motivational factors may influence SoA via multiple pathways, including indirect effects mediated by prospective fluency and retrospective outcome interpretation/causal inference. Therefore, it is necessary to distinguish findings based on the measures used (e.g., IB vs. agency ratings) when organizing this literature [8].

Additional research remains warranted to address the following gaps: first, the extent to which IB and sensory attenuation are specific markers of SoA remains debated, and it is necessary to dissociate contributions from general processes, including causal and time perceptions [39,77,78,79]. Second, a key challenge is how to experimentally manipulate and estimate the precision (reliability) of internal and external cues and identify context-dependent and individual-difference variations in weighting within computational models [8,41]. Third, it remains unclear how to provide a unified computational explanation for the dynamic reconfiguration of the relative contributions of FoA/JoA and prospective/retrospective cues across tasks, learning, and contexts [7,42,53,67].

Taken together, these perspectives suggest that SoA is best understood as a multi-stage, context-sensitive, and dynamically updated process, rather than the output of a single mechanism. In the present review, this synthesis is used to emphasize three analytically separable but interacting dimensions: (i) the relative contribution of prospective cues, retrospective cues, and their dynamic interaction; (ii) the distinction between prereflective FoA and reflective JoA; and (iii) the candidate contribution of WM-related processes to the maintenance of predictive representations, the comparison between predicted and actual outcomes, and the context-sensitive updating of cue weights. From this perspective, aging-related differences in SoA are unlikely to reflect a single linear decline, but instead arise from distinct changes in these component processes and their interactions. The following sections are organized according to this framework to review SoA measurement, aging-related variation, and the possible role of WM-related processes.

## 4. Methods for Measuring SoA

The measurement of the SoA has developed against the backdrop of two broad perspectives: one that treats volitional action (volition) as a central precondition for SoA, and another that more broadly focuses on the causal linkage between action and outcome and/or self-attribution (i.e., attributing an outcome to oneself), rather than on volition per se [1]. Consistent with the latter perspective, some studies have attenuated the volitional component using conditions, including passive movement [80], externally induced movement [77,81], and action observation [82,83,84], thereby examining the extent to which people feel or judge that they are the cause even when volitional involvement is relatively weak. This section categorizes SoA measures into explicit and implicit approaches, and briefly discusses how to interpret differences across measurement families. For each of these measurement families, we summarize key advantages, limitations, and interpretive caveats. Here, we use volition to refer to self-initiated action selection and motor intention, whereas agency denotes the experience and/or judgment that an outcome was caused by oneself. Therefore, paradigms that attenuate volition (e.g., passive or externally induced movement, and action observation) are best interpreted as probing attributional and postdictive components of agency, rather than as demonstrating intact volitional SoA in the absence of intention.

For example, Minohara et al. [10] assessed both explicit and implicit measures using a switch-press task and introduced a delay between the action (button press) and its outcome (stimulus change). This paradigm enables a direct comparison of whether increasing action–outcome delays exerts similar or dissociable effects on subjective agency reports and implicit measures. Such designs provide a useful basis for discussing relationships across measures, because the same manipulation (delay) can be evaluated across multiple measures in parallel.

### 4.1. Explicit Measures of SoA

Explicit SoA typically requires participants, during or after the task, to answer questions, including “Did you cause the change?” or “To what extent did you feel that your action produced the outcome?” Responses are frequently collected as a dichotomous Yes/No judgment [10] or as proportional/continuous ratings [85]. This approach is intuitive, easy to implement, and can capture subjective changes directly in response to experimental manipulations (e.g., temporal delay). However, explicit reports are vulnerable to demand characteristics (inferring the researcher’s intent), post hoc inference (rationalizing “I did it” after seeing the outcome), and contamination by expectations, beliefs, and question framing. Accordingly, explicit measures may reflect not only lived experience but also higher-order judgment processes [86]. Therefore, when using explicit measures, it is desirable to standardize rating timing and question format, include manipulation checks, and—when possible—combine explicit measures with implicit measures to strengthen interpretive robustness.

### 4.2. Implicit Timing-Based Measures of SoA

**Figure 1:**
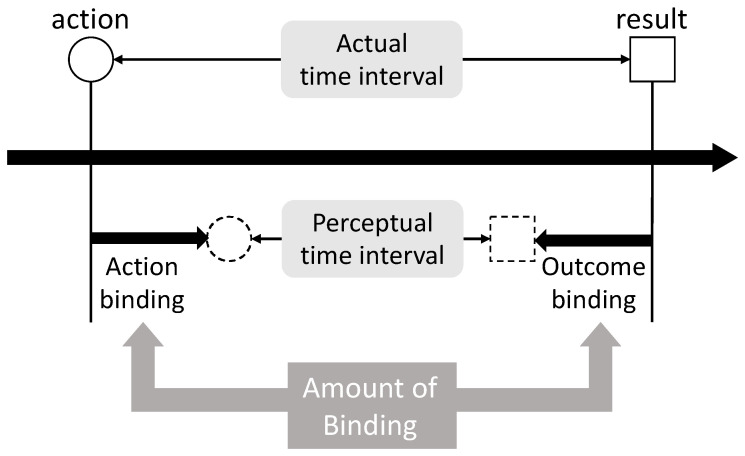
Intentional binding.

IB, a representative implicit measure, refers to the phenomenon in which the subjectively perceived interval between an action and its outcome is compressed relative to the actual temporal interval [38]. It has been used as an index of the strength of action–outcome coupling. IB is frequently discussed as comprising two components: action binding (the perceived time of action is shifted later) and outcome binding (the perceived time of the outcome is shifted earlier) [41,56] (see Moore et al. [87] for a review) (Figure 1). Although IB can capture one aspect of SoA, it can also vary due to factors other than volition (as discussed below). Therefore, caution is required when treating IB as a specific marker of SoA per se.

Haggard et al. [38] reported that when voluntary actions produce outcomes, perceived times of actions and outcomes shift toward each other, and they positioned this effect as an implicit index related to SoA. The authors also observed that IB is weaker for movements induced externally (e.g., via electrical stimulation) than for voluntary actions [38]. In contrast, other studies suggest that IB can occur even when volitional action is not clearly present [78,88]. For instance, Buehner et al. [78] showed that IB-like effects can arise (albeit more weakly) not only for one’s own actions but also during the observation of another person’s actions. Collectively, these findings indicate that IB is not uniquely determined by volition alone; rather, it may be influenced by multiple processes, including causal inference, prediction, attention, and contextual factors. Therefore, when using IB as an implicit measure of SoA, it is important not to equate “changes in IB” with “changes in SoA” in a simplistic manner, but instead to specify which factors (e.g., volition, causal inference, and contextual manipulation) are being manipulated and interpret results accordingly.

### 4.3. Interpreting Divergence Across Measurement Families

Explicit and implicit measures do not necessarily reflect the same underlying processes, and weak correlations between them have been reported [39,89]. For example, Ebert et al. suggested that longer action–outcome delays reduce the consistency between explicit and implicit measures. Such weak associations do not automatically imply that either measure is invalid; rather, they may indicate that SoA includes at least (i) a prereflective level of action–outcome coupling (implicit) and (ii) a reportable, self-attributional level of judgment (explicit). Therefore, depending on the research goal, it is essential to clarify whether the study targets subjective self-attribution judgments or action–outcome coupling, and, when feasible, to use both measures to improve interpretive validity [39,89].

Recently, beyond behavioral measures, there has been increasing use of neuroimaging techniques (functional magnetic resonance imaging (fMRI) [90,91]) and electrophysiological methods (electroencephalography (EEG) [13,92,93]), as well as immersive VR paradigms [91,94], to evaluate SoA in contexts with greater ecological validity. These approaches may help identify which processing stages underlie divergences between explicit and implicit measures (e.g., prediction generation, error processing, causal inference, and contextual integration), and align well with this review’s focus on the roles of WM and aging. Moving forward, frameworks that combine multiple measures (subjective ratings + IB + neural/physiological measures) will be increasingly important for decomposing SoA into component processes and discussing their respective contributions.

Collectively, these measurement-related considerations suggest that the paradigms reviewed here should not be considered as interchangeable indicators of a single latent agency construct. Instead, different measurement families likely capture partially overlapping, but non-identical, components of agency, with some paradigms emphasizing action–outcome coupling while others reflecting reportable self-attribution or evaluative judgment. This point is consistent with the two-step account, which distinguishes prereflective FoA from reflective JoA, thereby providing a framework for understanding why different paradigms emphasize different stages of agency processing [42]. It also aligns with empirical results showing that implicit and explicit measures do not necessarily converge, implying that dissociations between measures may reflect theoretically meaningful differences rather than mere methodological noise [39]. Therefore, the possible contribution of WM-related processes should be interpreted in a measure-sensitive manner, rather than assuming that all agency paradigms index the same underlying process.

Compared with the actual time interval between an action and its outcome, the perceptual time interval is experienced as shorter (temporal compression). The shift in the perceived timing of the action toward the outcome is termed action binding, whereas the shift in the perceived timing of the outcome toward the action is known as outcome binding. The amount of binding conceptually represents the magnitude of these shifts (or the reduction in the perceived interval).

## 5. SoA and Age-Related Changes

Across the lifespan, age differences in the SoA have been reported for both explicit and implicit measures. However, the available evidence remains limited, and substantial heterogeneity exists in tasks, outcome measures, and sampled age ranges. Accordingly, caution is warranted in reducing observed age-related differences to a single underlying mechanism.

First, regarding IB as an implicit index, it is typically observed in healthy adults, whereas studies in children and older adults suggest that IB is attenuated or varies in a condition-dependent manner [21]. For example, Fujii et al. compared voluntary and involuntary actions while manipulating the effector (hand vs. foot). They reported that older adults showed temporal compression comparable to that of younger adults in the voluntary condition, while exhibiting pronounced temporal compression even in the involuntary condition [95]. This pattern suggests that IB is not determined solely by intentionality, but can also be shaped by causal beliefs and task demands. Importantly, lifespan studies frequently rely on relatively small samples (approximately 20 participants per group), and older-adult samples typically vary widely in age range and health status (sensory, motor, and cognitive functioning), which should be explicitly considered when interpreting group differences. Moreover, estimates of IB depend strongly on analytic and design choices, including how baseline conditions are defined, whether action and outcome binding are analyzed separately, and whether the task uses temporal-order judgments versus interval estimation. Therefore, age-related differences should be evaluated regarding component processes—including temporal sensitivity, recalibration, and response strategies—rather than treated as a unitary change in SoA. Additionally, although IB may relate to SoA, it is also influenced by factors such as time perception, attentional allocation, motor preparation, and sensory thresholds; therefore, it should be interpreted cautiously as a stand-alone indicator of changes in SoA [87].

Studies based on explicit measures have reported that, in tasks manipulating external cues, older adults tend to show relatively smaller modulation of agency ratings by those external cues [22,24]. This pattern has been interpreted as reflecting a relative shift in older adults’ agency judgments toward greater reliance on internal cues. Additionally, older adults’ SoA has been reported to be less sensitive to manipulations of action–outcome delay [23], and recent research suggests that the balance of cues used to form agency judgments differs between older and younger adults [96]. However, it would be premature to collapse these findings into a single linear account (i.e., “reduced sensitivity to external cues → increased reliance on internal predictions”). Aging is accompanied by broad multi-system changes—including slower processing speed, declines in executive functions such as attention and inhibition, reduced sensory precision, and increased motor variability—that can alter task performance and the dynamics of subjective ratings [97,98,99,100,101]. Indeed, sensorimotor temporal integration and recalibration may change with age [102]. Accordingly, apparent “insensitivity” to IB or delay manipulations may be explained not only by shifts in cue weighting, but also by constraints in temporal resolution and available attentional resources.

Collectively, age-related changes in SoA are more plausibly understood as the outcome of multiple interacting pathways: (i) reduced reliability of sensory signals and increased noise, (ii) altered updating of internal models/predictions, (iii) declines in executive control (e.g., attention, inhibition, and action selection), and (iv) age-related modulation of frontostriatal and frontoparietal networks that support these functions [100,103].

### 5.1. Methodological Challenges in Aging Research

Another methodological concern in this literature is the significant variability among older adult samples. Variability in cognitive reserve, sensory function, motor variability, comorbidity, and medication status should be considered when interpreting age-related differences in SoA, as all of these factors influence agency-related performance and WM-related measures. In addition, information-processing speed, sensory thresholds, and general medical burden or overall health status should be carefully assessed or controlled, as apparent age effects may instead reflect broader age-associated changes rather than specific alterations in agency-related processing. These considerations are particularly important when sample sizes are small, age ranges are broad, and studies rely on a single measurement paradigm, since under such conditions, apparent age effects may be especially difficult to attribute to agency-specific mechanisms.

### 5.2. Future Directions for Aging Research

Considering these methodological challenges, future studies should go beyond accumulating cross-sectional findings by adopting longitudinal designs that track explicit and implicit SoA measures alongside sensory thresholds, attention/inhibitory control, WM, and motor variability within the same individuals. Additionally, transdiagnostic comparisons across neurodegenerative conditions (e.g., mild cognitive impairment/Alzheimer’s disease, Parkinson’s disease/dementia with Lewy bodies, and frontotemporal dementia) may help determine whether SoA alterations reflect general aging processes or dysfunction in specific circuits (e.g., dopaminergic and frontoparietal systems) [104]. Such studies should also measure and control key confounding factors, including medication status, depression/apathy, motor symptoms, and sensory impairment.

## 6. Relationship Between WM and SoA

WM is a capacity-limited system that temporarily maintains goal-relevant information and supports its manipulation and updating through multiple subsystems [25,105]. WM performance declines with age [33,34,106]. Rieck et al. [106] reported that older adults (aged 55–69 years) show reduced modulation of brain activity as task difficulty increases, and that performance becomes more vulnerable under higher cognitive demands. More generally, human cognitive resources are capacity-limited [107]; therefore, age-related reductions in available resources may leave less capacity to maintain predictive representations and integrate multiple sources of information, even when performing the same task.

WM impairments have also been reported across diverse clinical conditions, including schizophrenia [108,109,110,111,112], Parkinson’s disease [113,114], and depression [115]. The key point here is not disorder-specific symptomatology, but the general observation that reduced WM function can propagate to higher-order processes, including attention, decision-making, and learning. In turn, WM limitations may affect agency-related processes that operate on predictions about action outcomes and subsequent sensory feedback. Within cue-integration accounts, the SoA arises from comparing and integrating internally generated predictions (internal cues) with post-action sensory feedback and contextual information (external cues) in a context-sensitive manner [8]. From this perspective, maintaining predictive representations, performing prediction–outcome comparison, and updating context-dependent cue weights are likely to rely on WM and domain-general cognitive control resources.

Recently, this hypothesis has received behavioral support from dual-task paradigms that directly manipulate WM load. Under high WM load, explicit agency ratings decrease [29], and the implicit SoA indexed by IB can be attenuated by cognitive load or effort-related depletion [27]. Moreover, during continuous visuomotor control, increasing memory set size reduces perceived agency as measured by control ratings [116]. Collectively, these findings suggest that SoA is not merely a retrospective judgment but may depend on the stability of online processes that maintain predictive representations and support action–outcome comparison.

Importantly, “WM load” in dual-task settings can recruit domain-general cognitive control resources beyond WM per se, including attentional allocation and executive control.

Wen et al. [28] used a dual-task continuous-control paradigm in which participants controlled a moving dot while simultaneously maintaining digits in working memory; in the cued-goal-directed condition, they were instructed to move the dot toward a specific externally presented destination, and feedback on goal attainment was provided. The authors found that SoA decreased under high load when goal cues were absent, whereas the effect of load was smaller when such external goal-related cues were available. This pattern is consistent with the idea that SoA is shaped by the relative reliability of internal predictions and external cues: higher load may weaken comparison processes based on internal cues, thereby increasing reliance on external cues in some contexts. However, this shift cannot be uniquely attributed to WM impairment, as it may also reflect changes in attentional allocation and executive control; therefore, causal interpretation requires caution. Accordingly, Section 8 provides a more detailed review of how cognitive/WM load manipulations modulate explicit and implicit measures of SoA and discusses paradigm differences and alternative interpretations (e.g., attentional shifts).

**Figure 2:**
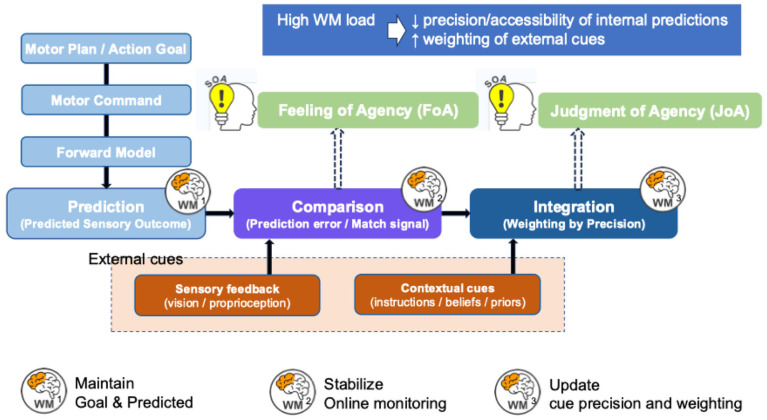
Sense of Agency and Working Memory.

Based on these considerations, Figure 2 schematically summarizes the core processes involved in SoA formation and the processing points at which WM may contribute, namely maintaining predictive representations, enabling comparison, and supporting context-dependent updating of cue weights. Conceptually, WM may support SoA at least at three processing points. First, WM can maintain an action intention and a predictive representation of the expected outcome until sensory feedback becomes available, enabling prediction–outcome comparison within a temporal window. Second, WM can maintain recent feedback and task context for longer, supporting evaluations based on action–outcome correspondence (or prediction error) rather than purely retrospective impressions. Third, WM can maintain context-dependent information regarding cue reliability (e.g., how informative goal cues or feedback are in a given task), supporting the updating of cue weights. Under high load, these operations may become less stable, potentially reducing effective use of internal predictions and increasing relative reliance on salient external cues [28,29,116], consistent with cue-integration frameworks [8].

As illustrated in Figure 2, SoA formation involves constructing and maintaining predictive representations before action, comparing them with post-action sensory feedback, evaluating discrepancies, and updating cue weights in a context-sensitive manner [8]. If these processes are constrained by WM load and associated cognitive control resources, WM and SoA may be linked at the behavioral level and through shared or interacting neural substrates. Therefore, the next section summarizes the neural bases supporting comparison/integration processes implicated in SoA and maintenance/updating processes associated with WM, and organizes candidate mechanisms through which these systems may intersect.

Figure 2 summarizes a candidate process-level account linking SoA and WM by integrating a two-step account of agency with precision-weighted cue integration. From the motor plan/action goal, a motor command is generated, and a forward model predicts the sensory outcome (Prediction). This predicted outcome is compared with sensory feedback, yielding a prediction error or match signal (Comparison). Contextual cues (e.g., instructions, beliefs, and priors) are combined with comparison-related information during a subsequent integration stage (Integration), yielding a final estimate of agency. In this framework, comparison-related processing is hypothesized to contribute primarily to the prereflective FoA, whereas integration-related processing contributes primarily to the reflective JoA. Solid arrows indicate the principal flow of action-related information. Dotted arrows indicate hypothesized conceptual links between lower-level processing stages and higher-level agency components, rather than direct proof of a one-to-one causal mechanism. WM1–WM3 denote three candidate points at which WM-related and executive-control processes may contribute: WM1, maintenance of action goals and predicted representations before feedback; WM2, stabilization of online monitoring required for prediction–feedback comparison; and WM3, maintenance and updating of cue reliability (precision) during context-sensitive integration. The orange boxes denote external cues, including sensory feedback and contextual cues. The top panel summarizes the hypothesized effect of high WM load: reduced precision or accessibility of internal predictions and increased relative weighting of external cues. This figure should be interpreted as a conceptual summary of candidate processing points, not as evidence for a single linear or anatomically localized mechanism.

## 7. Neural Mechanisms Linking SoA and WM: A Process-Specific Account

Neural activity associated with SoA has been widely reported in studies using fMRI and positron emission tomography [12,117], as well as EEG [13]. Candidate regions include frontal areas (including the pre-SMA) [118,119,120,121,122,123], the parietal cortex—such as the angular gyrus [66,90,91,124] and the temporoparietal junction (TPJ) [117,124]—and the cerebellum [118,125,126], among others [127].

However, presenting these findings merely as a list of “associated regions” can obscure the neural mechanisms that generate SoA. Currently, it may be more appropriate to conceptualize SoA not as a phenomenon reducible to a single neural mechanism, but as an emergent product of multiple action–outcome processes operating in coordination [127]. Therefore, in this review, we frame SoA as a network-level phenomenon, supported by interacting processes involving action and its consequences. Accordingly, the following discussion is organized around process-specific mappings rather than anatomical overlap: action selection/intention formation, prediction maintenance, prediction–outcome comparison, cue integration, and explicit/metacognitive agency judgment.

### 7.1. Process-Specific Neural Mechanisms of SoA

For SoA in contexts involving overt or intended action, the neural evidence can be organized around at least five partially overlapping processing components: (i) action selection and intention formation, (ii) prediction maintenance, (iii) prediction–outcome comparison, (iv) cue integration, and (v) explicit or metacognitive agency judgment. Frontal regions have been suggested to play an important role in these processes. For example, IB changes following transcranial direct current stimulation (tDCS) targeting the pre-SMA [119], and tDCS targeting the dorsolateral prefrontal cortex (DLPFC) modulates IB [120]. Moreover, the DLPFC can be engaged by tasks that require response selection and the construction and maintenance of a response space [121,122]. Accordingly, in SoA paradigms that require action selection, the frontal cortex—particularly the DLPFC—may support the selection and maintenance of action-related information, which could influence SoA for outcomes through interactions with parietal regions [66,120,121,122]. These findings are most relevant to the action-selection and intention-formation components of SoA. In this context, the DLPFC should not be interpreted merely as a region shared by SoA and WM; rather, it may support the selection and maintenance of action goals or response spaces before sensory feedback becomes available. Importantly, evidence from non-invasive brain stimulation is constrained by limits in target specificity and reproducibility; outcomes may vary due to current spread, individual differences, and task dependence. Therefore, such findings may indicate causal involvement rather than establishing definitive causal mechanisms at this stage [128].

Prediction maintenance and prediction–outcome comparison are likely to depend on interactions among the premotor cortex, M1, SMA/pre-SMA, and the cerebellum [118,125,126]. Within forward-model accounts, the cerebellum is particularly relevant to the prediction and timing of sensory consequences, whereas frontal motor regions may contribute to the preparation and maintenance of action-related representations [126]. At the comparison stage, parietal regions, including the angular gyrus, TPJ, and inferior parietal cortex, are relevant because they have been linked to self–other attribution and the allocation of self versus external agency [124]. These regions may therefore contribute not only to detecting mismatches between predicted and actual outcomes, but also to integrating information about whether an outcome should be attributed to oneself or to an external source. Overall, these findings support a view in which SoA depends on coordinated activity across multiple regions, including frontoparietal and cerebellar contributions.

Additionally, neural correlates specifically related to agency judgment have been reported in prefrontal regions [129], and links between self-agency disturbances (e.g., in schizophrenia) and frontal mechanisms have been discussed [123]. This is important because it indicates that action generation/selection and metacognitive self-attribution of action outcomes may not be identical processes. Consequently, when discussing SoA, it is essential to clarify which stage is being addressed: action selection, outcome monitoring, and/or explicit judgment [66,129]. This distinction is central to the present review because WM-related processes are likely to contribute differently across these stages. For explicit or metacognitive agency judgment, WM may be particularly important for maintaining task rules, recent action–outcome information, and response criteria, thereby supporting reportable JoA rather than prereflective FoA alone.

WM is also implemented through distributed interactions among prefrontal, parietal, temporal, thalamic, and subcortical regions [130,131,132,133,134,135,136,137,138,139,140,141]. However, its relevance to SoA lies not in anatomical overlap per se, but in the operations these networks support: maintaining action goals and predicted outcomes, stabilizing prediction–feedback comparison, updating cue reliability, and supporting explicit/metacognitive judgment. Thus, WM-related neural systems should be interpreted as candidate contributors to specific computational components of agency rather than as evidence for a single shared “agency–WM region.”

Osaka et al. reported that, in a verbal WM task, the DLPFC may contribute to maintaining attention to task goals, the anterior cingulate cortex may aid in suppressing irrelevant information, and the superior parietal lobule may facilitate attentional switching [135]. This pattern suggests that WM is implemented through interactions between a DLPFC-centered system and broader attentional control networks, and underscores the potential relevance of frontoparietal networks in considering neural substrates that support SoA.

### 7.2. Dynamic Network Evidence from Recent Neuroimaging and Neurotechnology Studies

Recent neuroimaging and neurotechnology studies further support this process-specific account by showing that SoA depends on task-specific information flow and dynamic network interactions. For example, fMRI/MVPA evidence has shown that different regions represent different levels of agency-related information: the right supramarginal gyrus within the inferior parietal lobe was most sensitive to self–other attribution, whereas the bilateral precentral gyri and left IPL more strongly reflected sensorimotor information [142].

BMI studies are also particularly informative because they allow decoded motor intention, overt movement, and sensory feedback to be partially dissociated. Intracortical BMI evidence suggests that M1 encodes not only motor and sensory information, but also sensorimotor conflict and subjective agency states. This finding supports the view that M1 should not be treated merely as an output structure, but as part of a sensorimotor loop in which motor predictions, feedback, and agency-related signals are dynamically coordinated [143]. Complementary EEG-BMI evidence suggests that agency during BMI control is strongly influenced by controllability and feedback. Such findings are useful for separating agency-related processes from overt bodily movement and for clarifying how external feedback can dominate agency judgments when proprioceptive feedback is reduced or absent [144,145]. Recent EEG-BMI evidence further indicates that agency depends on temporally organized oscillatory and connectivity dynamics. In a BMI user, pre-movement low-alpha oscillations in M1 predicted agency judgments. In healthy participants using EEG-BMI, pre-movement alpha oscillations in M1 and SMA were associated with agency ratings and functional connectivity changes involving parietal, temporal, and prefrontal regions [92].

VR studies provide complementary evidence for contextual and inferential components of agency. After participants controlled an avatar hand in VR, passive observation of the avatar’s subsequent action increased temporal binding, and this binding effect was associated with the right angular gyrus and inferior parietal lobule. Such findings suggest that prior control experience can potentiate inferential processing in the right inferior parietal cortex and generate an illusory SoA even without voluntary action [91]. Neurofeedback studies further support the view that agency-related processing reflects modifiable network states. EEG-based neurofeedback has shown that individuals can self-modulate agency-related neural activity and improve sensory-guided behavioral control, whereas recent real-time fMRI neurofeedback targeting the right TPJ suggests that explicit SoA can be enhanced in patients with functional neurological disorder [13,146]. Together, these fMRI/MVPA, BMI, VR, EEG-connectivity, and neurofeedback findings support the view that SoA is not simply associated with isolated regional activations. Rather, it emerges from task-specific information flow and dynamic interactions among sensorimotor, parietal, cerebellar, and prefrontal systems.

### 7.3. Aging-Related Network Changes and Cue Weighting

This process-specific account is particularly important for understanding aging. Age-related changes in SoA should not be interpreted as a uniform decline in a single agency mechanism. Rather, aging may alter several network-level operations that support agency, including the precision of sensorimotor predictions, the temporal stability of prediction–outcome comparison, the updating of cue reliability, and explicit or metacognitive evaluation. Evidence from aging research suggests that older adults may rely more strongly on sensorimotor prediction, and that this shift is associated with structural and functional differences in the pre-SMA and frontostriatal connectivity. This provides a neural basis for the idea that aging can alter the balance between internal predictions and sensory evidence during agency-related processing [100]. In parallel, age-related changes in frontoparietal control networks and large-scale network organization may reduce the stability of WM-related maintenance and updating processes. Although such findings are not specific to SoA, network-control evidence indicates that aging is associated with reduced controllability in large-scale networks, particularly the frontoparietal control and default-mode networks. These changes may provide a plausible neural framework for understanding how aging-related reductions in cognitive control could influence cue integration and explicit agency judgment [147]. In addition, resting-state and large-scale network studies of healthy aging suggest that age-related alterations in structural–functional network organization, including changes in integration and segregation within prefrontal, sensorimotor, and cerebellar systems, may further constrain the flexible coordination required for prediction, comparison, and cue updating during agency-related processing [148].

Overall, SoA and WM depend on large-scale networks that include frontal, parietal, cerebellar, and subcortical systems. However, it is insufficient to conclude merely that “both involve the frontal lobe” or that they share a common anatomical substrate. Instead, future studies should specify which stage(s) of SoA are supported by WM-related processes, including the maintenance of intention and predicted outcomes, prediction–outcome comparison, cue integration, and explicit/metacognitive judgment, while testing these hypotheses within frontoparietal and cortico-subcortical circuit frameworks. In the next section, we build on this process-level framing to examine how WM load manipulations influence implicit measures such as IB and explicit measures, including agency judgment [27,29].

## 8. WM as a Cognitive Resource for the SoA

As discussed above, WM-related processes may contribute to the generation and maintenance of selected components of SoA. SoA can be understood as a process where predicted action outcomes are compared with actual sensory consequences and, when needed, integrated into a coherent experience of control. Accordingly, when cognitive resources that temporarily maintain, update, and preserve prediction- and outcome-related information in a comparable format—including WM and closely related attentional resources—are limited, SoA may become more variable. Importantly, many “cognitive load” manipulations in this literature rely on dual-task paradigms; therefore, they can simultaneously alter not only WM load but also attentional allocation and task strategies. Consequently, load-related effects should be interpreted cautiously, avoiding a one-to-one attribution to “pure WM” mechanisms.

Regarding explicit SoA, Hon et al. [29] reported that it decreased under high load when participants maintained a consonant list (low load: two letters; high load: six letters) while performing an SoA task. This indicates that constructing and maintaining predictive representations of action outcomes may depend on the availability of cognitive resources. Concurrently, such decreases can be explained by attentional shifts or strategic changes rather than WM capacity limits per se [149]. Therefore, the result should not be interpreted as direct evidence that reduced WM leads to diminished SoA without considering alternative accounts.

Turning to implicit SoA, Howard et al. [27] examined an interval-reproduction task where an outcome tone occurred 500–1500 ms after an initiating event (active button press vs. passive auditory trigger). The authors found that, in the active condition, IB decreased as cognitive load increased, whereas binding increased under low load. However, a critical caveat is that IB cannot be treated unreservedly as a pure proxy for SoA. First, time-estimation tasks are highly sensitive to attentional allocation and response strategies, and load manipulations may amplify these influences. Second, the set of confounding factors differs across intentional-binding paradigms (e.g., Libet clock tasks vs. interval reproduction/estimation); however, time judgment itself is generally susceptible to attentional and strategic factors. Consistent with this, in Libet clock paradigms, merely manipulating the distribution of visuospatial attention can systematically bias time reports and modulate temporal binding [150]. Third, although it has been hypothesized that binding in the Libet clock task is grounded in spatial WM processes, evidence remains inconsistent: one report found only limited correlational support and no decisive support from dual-task load manipulations [151].

Additionally, cognitive load does not necessarily shift explicit and implicit measures in the same direction, and dissociations across measures can occur. For example, WM load dissociates explicit and implicit measures of body ownership and agency [152]. Moreover, correlations between implicit measures of SoA (e.g., binding and sensory attenuation) and explicit agency ratings are frequently weak or inconsistent [39]. Thus, measurement limitations should be stated explicitly, and conclusions should not rest on any single index.

Whether cognitive load reliably reduces SoA also appears to be context dependent. Wen et al. [28] manipulated the presence of goal cues in a continuous control task in which participants controlled a moving dot while performing a concurrent memory task; in the cued-goal condition, the participants were asked to guide the dot toward a specific externally presented destination and received feedback on goal attainment. The authors found that higher memory load reduced SoA, particularly when goal cues were absent, whereas this effect was attenuated when external goal-related cues were available. This pattern suggests that even when internal cues are weakened, external cues (e.g., goals, contextual information, or outcome consistency) provide compensatory support. Similarly, WM load does not uniformly diminish agency-related inferences: effects can differ between goal- and priming-based inferences [153]. Furthermore, although cognitive load reduces SoA during continuous action [116], SoA in such tasks can depend strongly on delay and performance [85]. Consequently, apparent load effects may vary with task structure and the evaluation window (e.g., trial-level vs. block-level judgments, or dependence on recent trials) [154]. Overall, isolating the underlying mechanism—WM capacity limits vs. attentional allocation, fatigue/arousal, or strategy use—remains essential before claiming a causal contribution of WM.

Finally, the direction of influence may not be limited to WM → SoA. Stronger SoA is associated with enhanced WM retention [155], and stimuli experienced as self-generated may be remembered better (the “self-agency effect”) [156]. However, the mechanisms by which SoA might enhance memory (e.g., prioritized attention, reward/motivation, and self-referential processing) and the causal direction remain unresolved. The next section outlines research strategies to disentangle WM from attention and related factors, while evaluating explicit and implicit measures in parallel.

## 9. Discussion and Future Research Agenda

In this section, we present evidence on the relationship between SoA and WM, with particular attention to the possibility that WM-related processes contribute to maintaining motor predictions and integrating internal and external agency cues. Importantly, the present review does not support reducing the SoA–WM relationship to a single linear causal pathway (e.g., “reduced WM capacity or increased cognitive load leads to reduced SoA”). Rather, the magnitude and direction of the association are likely to vary depending on task demands (e.g., prediction precision, cue reliability, and temporal estimation requirements) as well as individual strategies. Moreover, although some studies suggest that SoA may facilitate WM processing and memory, other studies have reported null effects, indicating that the causal direction and mediating mechanisms linking SoA and WM remain unresolved [155,156,157].

Methodologically, cognitive-load manipulations can affect multiple factors beyond WM, including attentional allocation, arousal, affective state, and time perception. Accordingly, load-related changes in SoA should not be attributed directly to WM capacity limitations without careful consideration of these potential confounds.

In addition, although IB has been widely adopted as an implicit measure of SoA, time judgments in Libet clock paradigms can be influenced by visuospatial attention and prediction-related processes [158]. The effects of cognitive load are also not consistent across studies, and null findings, opposite-direction effects, and alternative accounts based on attentional or affective shifts remain plausible. Therefore, conclusions should not rest on a single index (e.g., IB) alone. Instead, future studies should explicitly evaluate convergence and divergence across implicit and explicit measures and clarify which components of agency-related processing—including prediction maintenance, prediction–outcome discrepancy evaluation, cue integration, and metacognitive judgment—are constrained under which conditions.

### 9.1. Methodological and Analytical Recommendations

To improve interpretive consistency in this literature, future studies should report their analytical assumptions and measurement choices more explicitly. In particular, researchers should clearly specify which component of agency a given paradigm is intended to probe and avoid treating different measures as interchangeable by default. When intentional binding is used, analytic decisions that strongly affect interpretation—such as baseline definition, whether action binding and outcome binding are analyzed separately, and the choice of temporal judgment paradigm—should be reported transparently. Likewise, studies using explicit ratings should standardize question format, rating timing, and manipulation checks as much as possible.

More broadly, data analysis should account for sources of variability that may otherwise obscure the interpretation of SoA-related effects. These include intra-individual variability, baseline performance, recent-trial dependence, and the possibility that the same experimental manipulation may affect explicit and implicit measures differently. Where feasible, converging evidence across multiple indices should be prioritized over conclusions based on a single measure. In studies examining WM-related contributions, analyses should also distinguish effects plausibly attributable to WM from those that may reflect attentional allocation, temporal processing, arousal, or strategy use. Implementing these practices will improve comparability across studies and strengthen the interpretability of findings in this heterogeneous field.

### 9.2. Future Research Priorities

Several priorities may be considered for future research. First, studies should dissociate the component(s) altered by “load” manipulations (e.g., WM maintenance/updating, attentional allocation, temporal estimation, and affective processes) and formalize their contributions to SoA in testable models. Second, the SoA assessment should combine IB with explicit agency ratings and complementary behavioral or physiological measures to examine convergent validity and context dependence within the same participants. Third, ecologically valid paradigms (e.g., VR or teleoperation) may be useful for quantifying how delay and uncertainty in action–feedback contingencies shape the SoA–WM relationship [19]. Fourth, to address aging-related changes, longitudinal designs should track within-individual trajectories of SoA (implicit and explicit), WM, sensory precision, and executive control in order to examine temporal ordering and potential causal pathways [23]. Fifth, multimodal neuroimaging and neurophysiological approaches may help identify which neural processes—within frontoparietal networks and related systems—mediate the links among prediction maintenance, cue integration, and SoA.

From a clinical perspective, an important next step will be to determine whether WM-targeted interventions are associated with measurable changes in SoA in populations with documented agency disturbances, including schizophrenia [14].

For example, randomized controlled trials using interventions expected to improve WM—such as cognitive remediation [159,160] and tDCS targeting the left DLPFC [161]—could assess both WM and SoA outcomes (e.g., IB alongside explicit agency ratings) before and after intervention. Mediation analyses may help evaluate whether improvements in WM account for changes in SoA, whereas moderation analyses may clarify whether intervention effects depend on task properties such as cue reliability, delay, and cognitive demands. Such designs would provide a stronger basis for evaluating the possible clinical relevance of WM-targeted approaches for SoA-related processes. In addition, combining these interventions with neuromodulation approaches reported to modulate SoA-related measures [59], as well as exploratory neurofeedback approaches targeting agency-related processing [13], may help clarify potential mechanisms linking WM and SoA.

Overall, the relationship between SoA and WM is best understood as an interaction among multiple processes, including prediction maintenance, cue integration, and metacognitive evaluation. Progress in this field will depend on diverse measurement, longitudinal, and interventional approaches that strengthen causal inference, and integrative tests of neural mechanisms, thereby supporting theoretical frameworks relevant to both aging and clinical populations.

## Figures and Tables

**Table 1:** Summary and critical appraisal of key empirical studies discussed in this review.

Author (Year)	Design/Focus	Participants	SoA Measure	Key Finding	Limitation	Ref.
Haggard et al. (2002)	Action–outcome timing task comparing voluntary and externally triggered actions	Healthy adults (25–54 years)	Intentional binding (implicit SoA)	Voluntary action increased temporal binding, supporting intentional binding as an implicit agency index	Foundational study, but not focused on aging or working memory; intentional binding is not a complete proxy for SoA	[38]
Dewey and Knoblich (2014)	Comparison of implicit and explicit agency measures	Healthy adults (mean age 24.10 years, SD 3.22)	Implicit and explicit SoA measures	Implicit and explicit measures did not necessarily capture the same aspect of agency	Important methodological caution when comparing results across paradigms	[39]
Nobusako et al. (2020)	Developmental comparison of the temporal window of SoA	School-age children (6–12 years) and younger adults (21–23 years)	Explicit SoA/temporal window of agency	The temporal window associated with SoA differed across age groups	Developmental rather than aging study; indirect relevance to older adulthood	[20]
Cavazzana and Begliomini (2017)	Lifespan comparison using voluntary, involuntary, and auditory conditions	Children (8–11 years), younger adults (22–30 years), and older adults (66–76 years)	Intentional binding	Agency-related binding differed across age groups, with adult participants showing the clearest binding in the voluntary condition	Small group sizes and strong paradigm dependence limit generalization	[21]
Metcalfe et al. (2010)	Lifespan comparison of metacognitive aspects of agency	College students (18–24 years), older adults (mean age 78.47 years), and children (8–10 years)	Explicit/metacognitive agency judgment	Reported age-related differences in metacognitive aspects of agency	Explicit metacognitive judgment is not equivalent to implicit agency; not a direct working-memory test	[24]
Cioffi et al. (2017)	Vicarious-agency paradigm manipulating external cues	Experiment 1: younger adults (17–34 years) and older adults (54–72 years); Experiment 2: younger adults (18–35 years) and older adults (62–92 years)	Explicit SoA	Older adults appeared less susceptible to externally manipulated agency cues than younger adults	One specific paradigm; findings may also reflect age-related differences in attention or response strategy	[22]
Mariano et al. (2024)	Cross-sectional comparison with action–outcome delay manipulation	Thirty younger adults (mean age 22.0 years, SD 1.8) and thirty older adults (mean age 59.5 years, SD 4.9)	Explicit SoA and intentional binding	Older adults showed reduced explicit SoA and intentional binding, and their judgments were less affected by delay	Cross-sectional design; effects may reflect temporal resolution or attention, not only cue weighting	[23]
Howard et al. (2016)	Effort manipulation during an agency task	Healthy adults (18–29 years; mean age 20.39 years, SD 2.05)	Intentional binding (implicit SoA)	Physical and mental effort disrupted the implicit sense of agency	Working memory was indexed indirectly; intentional binding is also sensitive to timing and attention	[27]
Wen et al. (2016)	Divided-attention manipulation during agency processing	Healthy adults (22–33 years; mean age 25.7 years, SD 3.6)	Agency-related processing under divided attention	Divided attention altered processes underlying SoA	Focuses on attention more than working memory per se	[28]
Hon et al. (2013)	Dual-task manipulation of cognitive load during an agency task	Twenty-four undergraduate students (age not reported)	Explicit SoA	Higher cognitive load reduced explicit SoA	Working memory demand was manipulated indirectly through load; not an aging study	[29]
Miyawaki and Morioka (2020)	Self–other attribution task examining cognitive and sensorimotor cues	Twenty-one healthy right-handed volunteers (mean age 26.7 years, SD 7.7)	Agency attribution judgment	Cognitive and sensorimotor cues could dissociate and interfere with agency attribution	Not an aging study; task-specific self–other attribution paradigm	[40]
Wolpe et al. (2013)	Cue-integration manipulation in intentional binding	Healthy adults (18–36 years; mean age 26 years, SD 6)	Intentional binding	Agency-related timing judgments depended on cue integration rather than a single comparator signal alone	Mechanistic rather than direct aging or working-memory evidence	[41]

## Data Availability

No new data were generated. All data used in this study were obtained from publicly available sources or previously published literature.

## Abbreviations

The following abbreviations are used in this manuscript:

SoA	Sense of Agency
WM	Working Memory
fMRI	Functional Magnetic Resonance Imaging
EEG	Electroencephalography
TPJ	Temporoparietal Junction
tDCS	Transcranial Direct Current Stimulation
DLPFC	Dorsolateral Prefrontal Cortex
IB	Intentional Binding
SMA	Supplementary motor area
FoA	Feeling of Agency
JoA	Judgment of Agency
